# A County-Level Spatial Study of Serum Selenoprotein P and Keshan Disease

**DOI:** 10.3389/fnut.2022.827093

**Published:** 2022-01-28

**Authors:** Yuehui Jia, Ruixiang Wang, Shengqi Su, Lei Qi, Yuanyuan Wang, Yanan Wang, Yuanjie Zou, Xu Liu, Yiyi Zhang, Jie Hou, Hongqi Feng, Qi Li, Tong Wang

**Affiliations:** ^1^Institute of Keshan Disease, Chinese Center for Endemic Disease Control, Harbin Medical University, Harbin, China; ^2^School of Public Health and Management, Binzhou Medical University, Yantai, China; ^3^Ningbo Municipal Center for Disease Control and Prevention, Ningbo, China; ^4^Yidu Central Hospital of Weifang, Weifang, China; ^5^Yantai Disease Prevention and Control Center, Yantai, China; ^6^Third Affiliated Hospital of Harbin Medical University, Harbin, China

**Keywords:** Keshan disease, selenoprotein P, spatial epidemiology, elimination assessment, precision prevention and control

## Abstract

**Background:**

Keshan disease (KD) is strongly associated with selenium deficiency. Selenoprotein P (SELENOP) is a recognized molecular biomarker of selenoproteins and an important indicator of selenium nutrition. This study was aimed at providing geographically precisely visualized evidence of selenium nutrition at molecular level for assessing KD prevention, control, and elimination on the etiological perspective.

**Methods:**

We used spatial ecological design for this study. The serum SELENOP levels of the residents were measured by ELISA. ArcGIS version 9.0 was used for spatial description, spatial autocorrelation analysis of SELENOP levels and spatial regression with per capita disposable income.

**Results:**

The mean serum SELENOP levels of the 6,382 residents in 1,688 counties were 4.62 ± 1.82 μg/mL. The mean serum SELENOP levels of the residents living in the townships and rural areas of KD endemic counties were not statistically significantly lower than those of the KD non-endemic counties. The mean serum SELENOP levels were globally clustered (Moran's *I* = 0.03, *z* = 6.37, and *P* < 0.0001), and 99.3% (553/557) of the cold spots, identified by local autocorrelation analysis (Getis-Ord-Gi^*^ analysis), were located in the KD endemic provinces of Shaanxi, Shanxi, Henan, Hebei, Shandong, Inner Mongolia, Gansu, Hubei, Chongqing, Yunnan, and Sichuan. The serum SELENOP level was positively correlated with per capita disposable income (*t* = 3.52, *P* = 0.0004).

**Conclusions:**

The results of this study were the geographically precisely visualized evidence of selenium nutrition at molecular level for assessing KD elimination on the etiological perspective. The cold spot counties found by Getis-Ord-Gi^*^ analysis in the KD endemic provinces should be the high priority of KD precision prevention and control.

## Introduction

Keshan disease (KD) is a primary endemic cardiomyopathy that only occurs in low-selenium areas from the northeast to the southwest in mainland China (1–3). Although KD has been effectively controlled in most endemic areas, new cases of chronic Keshan disease (CKD) and latent Keshan disease (LKD) still exist in some endemic areas with poor economic conditions (4).

The goals of national KD prevention and control programs are elimination of Keshan disease (5, 6). In assessing the effectiveness of KD prevention, control, and elimination, the incidence or prevalence is the most important indicator. However, the indicators of etiology or risks are better in terms of primary prevention. The evidence of low selenium is the strongest in the KD etiology. Observational epidemiological studies showed that the KD endemic areas and the low selenium zone are overlapped, and the external and internal environments of the residents in KD endemic areas were selenium deficient (7–9). The interventional studies showed that selenium supplementation can effectively prevent the occurrence of acute and subacute KD (10–13). Therefore, the selenium levels are important etiological evidence for assessing the effectiveness of KD prevention, control, and elimination. Selenium in the human body plays biological roles in the form of selenoproteins (14–16). Selenoprotein P (SELENOP), accounting for about 60% of plasma selenium, is a secretory protein that contains multiple selenocysteine residues per polypeptide which acts as the primary transporter of selenium (17, 18). In selenium supplementation studies, SELENOP concentration increased over a wider range of selenium intakes than glutathione peroxidases (GPX3) (19, 20). Moreover, the serum selenium was found to be linearly correlated with SELENOP, but not with GPX3 activity (19). Thus, SELENOP is a recognized molecular biomarker of selenoproteins and an important indicator of selenium nutrition (21–23).

Spatial epidemiological study is mainly used to comprehensively describe and analyze the characteristics and clustering of diseases with geographic information system to visualize the spatial distribution of diseases. The outputs of spatial study are the evidence for prioritizing the keys of prevention and control, and assessing the effectiveness of prevention and control (24–26). Keshan disease is very suitable for spatial analysis because of its endemicity. Although we reported a study of serum SELENOP and KD at the provincial level (27), there has been no report on nationwide county-level spatial epidemiological study of serum SELENOP and KD in China. County-level spatial study is small area study. The outcomes of small area study are more reliable and geographically precise because of its small size of spatial study units. We therefore conducted this spatial epidemiological study of SELENOP and KD in order to assess the effectiveness of KD prevention, control, and elimination at county-level on the etiological aspects and provide geographically precise and visualized evidence of selenium nutrition at the molecular level for KD precision prevention and control on the etiological perspective.

## Materials and Methods

### Study Design

A spatial ecological study was designed to conduct a nationwide investigation of serum SELENOP at the county-level to investigate the serum SELENOP levels of residents living in KD endemic and non-endemic counties in mainland China, and to provide geographically precise and visualized evidence of selenium nutrition at the molecular level for KD prevention, control, and elimination on the etiological perspective.

The studies involving human participants were reviewed and approved by the ethics committee of the Harbin Medical University (hrbmuecdc20150101). This study was performed in accordance with the ethical standards as laid down in the 1964 Declaration of Helsinki and its later amendments or comparable ethical standards.

### Study Participants

**Figure 1:**
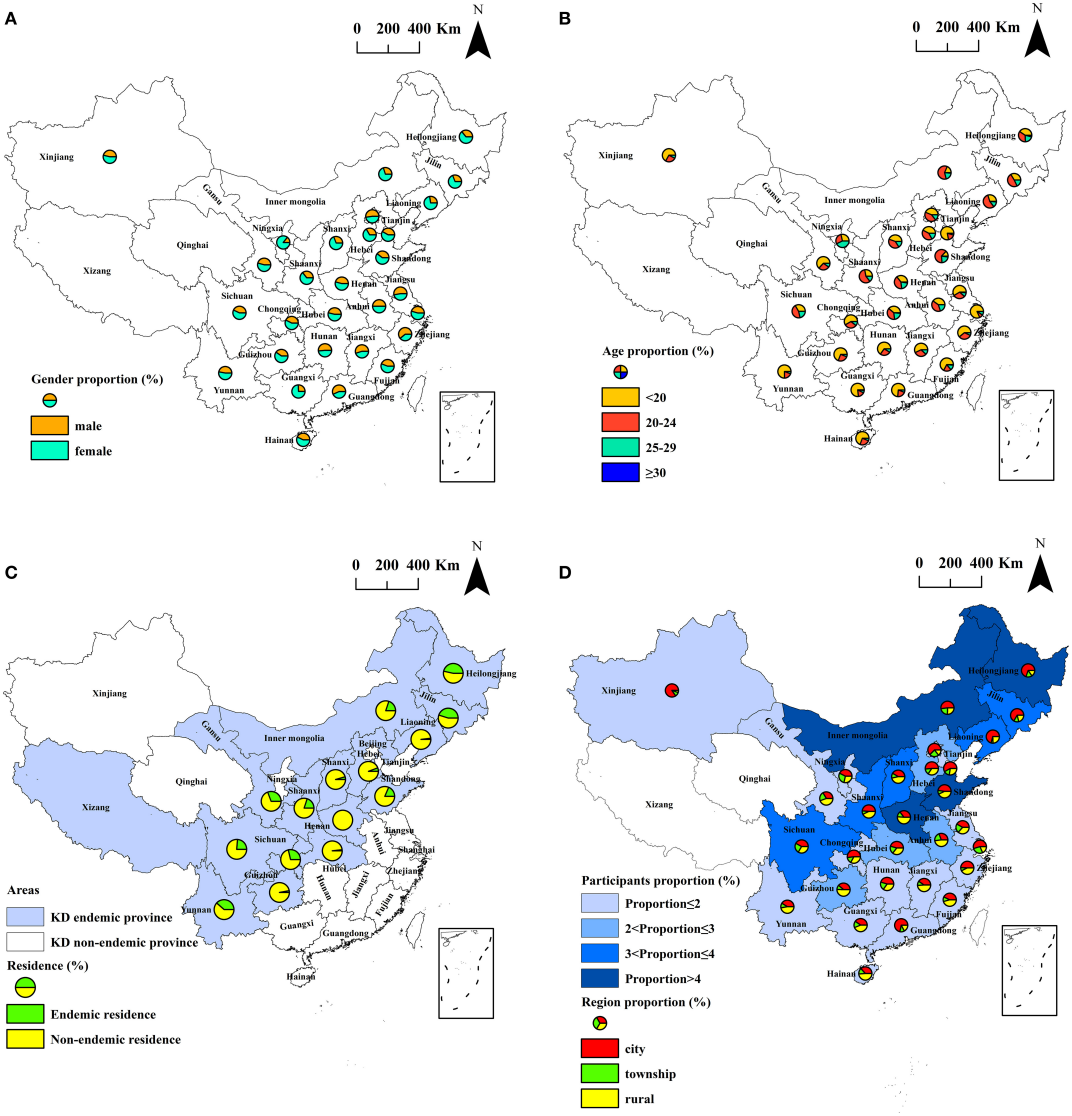
Spatial distribution of the subjects investigated. **(A)** spatial distribution of the gender of the subjects; **(B)** spatial distribution of the age of the subjects; **(C)** spatial distribution of subjects living in KD endemic areas and non-endemic areas; **(D)** spatial distribution of participants proportion and region proportion in different provinces.

During 2019–2020, 6,382 individuals aged 15–44 years, who had lived in their residence for not <6 months in the past 12 months, participated in the study. A total of 2,462 (38.6%) males and 3,920 (61.4%) females were surveyed. Among them, 2,639 (41.3%) participants were younger than 20 years old, 2,504 (39.2%) participants aged 20–24 years old, 1,101 (17.3%) participants aged 25–29 years old, and 138 (2.2%) participants were older than 30 years old. Of the participants, 3,605 (56.5%) lived in cities, 938 (14.7%) lived in townships, and 1,839 (28.8%) lived in rural areas. There were 4,758 (74.6%) participants from the KD non-endemic counties and 1,624 (25.4%) participants from the KD endemic counties. The participants were recruited from 1,688 counties in 29 provinces, and covered 59.4% (1,688/2,843) of all counties in mainland China, including 236 KD endemic counties (71.5%, 236/330) and 1,452 KD non-endemic counties (57.8%, 1,452/2,513) in mainland China. The details of the demographic characteristics of the subjects by gender, age, region by KD endemic area are shown in Table 1 and Figures 1A–D. The counties of the participants were divided into KD endemic areas and KD non-endemic areas according to the Keshan Disease Endemic Area Definition and Classification (GB17020-2010) (4, 28). According to the Administrative Division of China, the residence of the participants were divided into rural, township, and city areas (29). The inclusion criteria of participants were age ≥15 years old, were permanent residents who had lived in their residence for more than 6 months in the past 12 months, were healthy in general physical examination, were able to communicate normally and to participate in the research voluntarily. All participants provided written informed consent.

**Table 1:** Serum SELENOP levels by demographic characteristics and association with KD endemic area (μg/mL).

**Characteristic**	**All**			**KD endemic counties**			**KD non-endemic counties**			**Statistics**	***P*-value**
	***n***	**SELENOP ($\bar{x}$ ± *s*)**	**95% CI**	***n***	**SELENOP ($\bar{x}$ ± *s*)**	**95% CI**	***n***	**SELENOP ($\bar{x}$ ± *s*)**	**95% CI**		
All	6,382	4.62 ± 1.82	4.58–4.67	1,624	4.54 ± 2.03	4.44–4.64	4,758	4.66 ± 1.74	4.61–4.70	*z* = 2.24	0.0250^*^
Gender										
Male	2,462	4.70 ± 1.80	4.63–4.77	529	4.65 ± 1.95	4.48–4.81	1,933	4.71 ± 1.76	4.64–4.79	*z* = 0.76	0.4502
Female	3,920	4.58 ± 1.83	4.52–4.63	1,095	4.48 ± 2.07	4.36–4.61	2,825	4.61 ± 1.73	4.55–4.68	*z* = 1.99	0.0465^*^
Statistics		*z* = 2.60			*z* = 1.52			*z* = 1.94			
*P*-value		*P* = 0.0093^*^			*P* = 0.1298			*P* = 0.0522			
Age (year)										
<20	2,639	4.67 ± 1.68	4.61–4.74	684	4.34 ± 1.97	4.19–4.48	1,955	4.79 ± 1.54	4.72–4.86	*z* = 6.16	<0.0001^*^
20–24	2,504	4.63 ± 1.90	4.55–4.70	654	4.74 ± 2.09	4.58–4.90	1,850	4.59 ± 1.82	4.51–4.67	*z* = 1.76	0.0792
25–29	1,101	4.58 ± 1.99	4.47–4.70	267	4.57 ± 2.02	4.33–4.82	834	4.59 ± 1.98	4.45–4.72	*z* = 0.08	0.9400
≥30	138	4.00 ± 1.68	3.72–4.28	19	4.33 ± 1.65	3.54–5.13	119	3.94 ± 1.68	3.64–4.25	*t* = 0.94	0.3473
Statistics		*F* = 6.30			*F* = 4.51			*F* = 12.04			
*P*-value		*P* = 0.0003^*^			*P* = 0.0037^*^			*P* < 0.0001^*^			
Region										
Cities	3,605	4.72 ± 1.93	4.66–4.78	757	4.57 ± 2.13	4.42–4.72	2,848	4.76 ± 1.87	4.69–4.83	*z* = 2.44	0.0148^*^
Townships	938	4.50 ± 1.74	4.39–4.61	395	4.58 ± 2.08	4.37–4.78	543	4.45 ± 1.43	4.33–4.57	*z* = 1.09	0.2762
Rural areas	1,839	4.50 ± 1.64	4.43–4.58	472	4.46 ± 1.81	4.29–4.62	1,367	4.52 ± 1.57	4.43–4.60	*z* = 0.69	0.4926
Statistics		*F* = 11.20			*F* = 0.52			*F* = 13.22			
*P*-value		*P* < 0.0001^*^			*P* = 0.5971			*P* < 0.0001^*^			

* *P < 0.05 and the difference between groups were statistically significant*.

### Questionnaire Survey

A pre-designed questionnaire was used to investigate demographic information on participants regarding gender, age, current residence and address.

### Blood Samples

After fasting for 8 h, 2 ml of venous blood samples from participants was collected into vacuum blood collection tubes without anticoagulant. Blood samples were naturally agglutinated for 30 min, and then were centrifuged at 3,000×g for 10 min. Serum was carefully and immediately collected into tubes without pyrogen and endotoxin, and stored at −80°C in the refrigerator until the assay.

### Serum SELENOP

The human SELENOP enzyme-linked immunosorbent assay kits (Jiangsu Kejing Biotechnology Co., Ltd, Jiangsu, China) were used to measure SELENOP concentration in the serum samples. The SELENOP ELISA kit includes a set of SELENOP standards for calibration. The optical density (OD) of the calibration standards and the serum samples of participants were simultaneously measured at a wavelength of 450 nm using the BioTek Cytation 3 MFD, then the standard curve was generated by plotting the OD value obtained for each of the SELENOP standard concentration on the vertical (Y) axis vs. the corresponding concentration on the horizontal (X) axis. Finally, the concentration of SELENOP in each serum sample was calculated using the standard curve equation. The sensitivity in this assay was 0.1 μg/ml. The coefficients of variation of intra- assay was <7% and the inter-assay was <12%.

### Economic Data

Economic data of per capita disposable income counties at the county-level in this study were obtained from the Province Statistical Yearbook 2019–2020.

### Statistical Analysis

Data entry was performed using Epi Info (version 3.5.1). Data cleaning and statistical analysis were conducted using SPSS (version 17.0). SELENOP concentration is expressed as mean and standard deviation ($\bar{x}\pm s$). The normality of SELENOP concentration was assessed with a Shapiro–Wilk test. The *z*-test, independent-sample *t*-test and one-way analysis of variance (ANOVA) were conducted to compare the SELENOP levels. The test level of α was 0.05 (two sided), and *P*–value <0.05 was considered statistically significant. Spatial regression analysis of serum SELENOP and per capita disposable income was conducted by ordinary least squares (OLS) using ArcGIS (version 9.0). Spatial description of study variables was performed by plotting the thematic maps using ArcGIS (version 9.0). Spatial autocorrelation analysis was conducted using ArcGIS (version 9.0). Moran's *I* was used for global spatial autocorrelation analysis to explore whether spatial clustering of serum SELENOP existed at the overall level. The values of Moran's *I* ranged from +1 to −1, indicated spatially random (Moran's *I* = 0), dispersed (Moran's *I* < 0) or clustered (Moran's *I* > 0). Getis-Ord-Gi^*^ statistic was used for local spatial autocorrelation analysis to determine the types and locations of spatial clustering of serum SELENOP. The “hot spots” were determined by the positive *z*-scores, indicated that the high values of the mean serum SELENOP levels were clustered in these areas. The “cold spots” were determined by the negative *z*-scores, indicated that the low values of the mean serum SELENOP levels were clustered in these areas. The corresponding *z* values of 90, 95, and 99% confidential intervals (CI) for the Getis-Ord Gi^*^ were ±1.65, ±1.96, and ± 2.58, respectively.

## Results

### Serum SELENOP Levels and KD

The mean serum SELENOP levels of residents living in the townships and rural areas of KD endemic counties were not statistically significantly lower than those of residents living in KD non-endemic counties (*z* = 1.09, *P* = 0.2762; *z* = 0.69, *P* = 0.4926), although the mean serum SELENOP levels in KD endemic counties were statistically significantly lower than KD non-endemic counties (*z* = 2.24, *P* = 0.0250). The mean serum SELENOP levels of residents living in cities of KD endemic counties were significantly lower than that of residents living in KD non-endemic counties (*z* = 2.44, *P* = 0.0148).

The mean serum SELENOP levels of females in KD endemic counties were significantly lower than that of females in KD non-endemic counties (*z* = 1.99, *P* = 0.0465). The mean serum SELENOP levels of participants younger than 20 years of age in KD endemic counties were significantly lower than that of participants in KD non-endemic counties (*z* = 6.16, *P* < 0.0001). The mean serum SELENOP levels of 1,624 participants living in KD endemic counties were 4.54 ± 2.03 μg/mL (95% CI: 4.44–4.64), and were significantly different between age groups (*F* = 4.51, *P* = 0.0037). The mean serum SELENOP levels of 4,758 participants living in KD non-endemic counties were 4.66 ± 1.74 μg/mL (95% CI: 4.61–4.70), and were significantly different between age groups (*F* = 12.04, *P* < 0.0001), and regions (*F* = 13.22, *P* < 0.0001).

The mean serum SELENOP levels of the 6,382 residents in 1,688 counties were 4.62 ± 1.82 μg/mL (95% CI: 4.58–4.67), and ranged from 0.97 to 11.22 μg/mL. The mean serum SELENOP levels of females were significantly lower than that of males (*z* = 2.60, *P* = 0.0093). The mean serum SELENOP levels were significantly different between age groups (*F* = 6.30, *P* = 0.0003). The mean serum SELENOP levels of residents living in rural areas and townships were significantly lower than those of residents living in cities (*F* = 11.20, *P* < 0.0001). The above results of serum SELENOP levels by demographic characteristics and KD endemic area are shown in Table 1.

**Figure 2:**
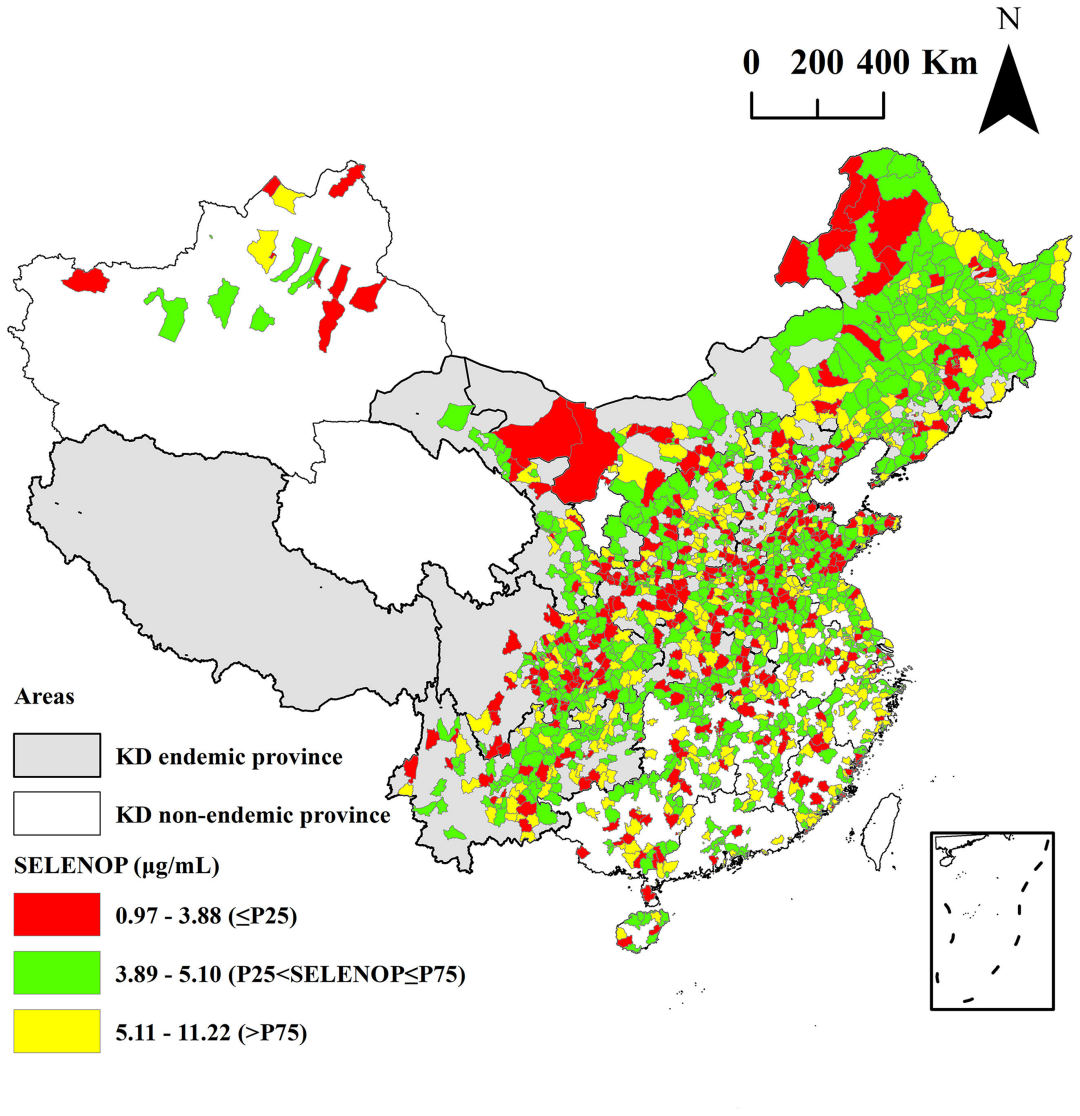
Spatial distribution of the mean serum SELENOP levels at the county-level.

The spatial distribution of the mean serum SELENOP levels in 1,688 counties are shown in Figure 2. The P_25_ and P_75_ value of the mean serum SELENOP levels in 1,688 counties were 3.88 and 5.10 μg/mL, respectively. A total of 417 counties of the mean serum SELENOP levels were lower than the P_25_ value.

### Spatial Regression Analysis of Serum SELENOP and Per Capita Disposable Income

**Figure 3:**
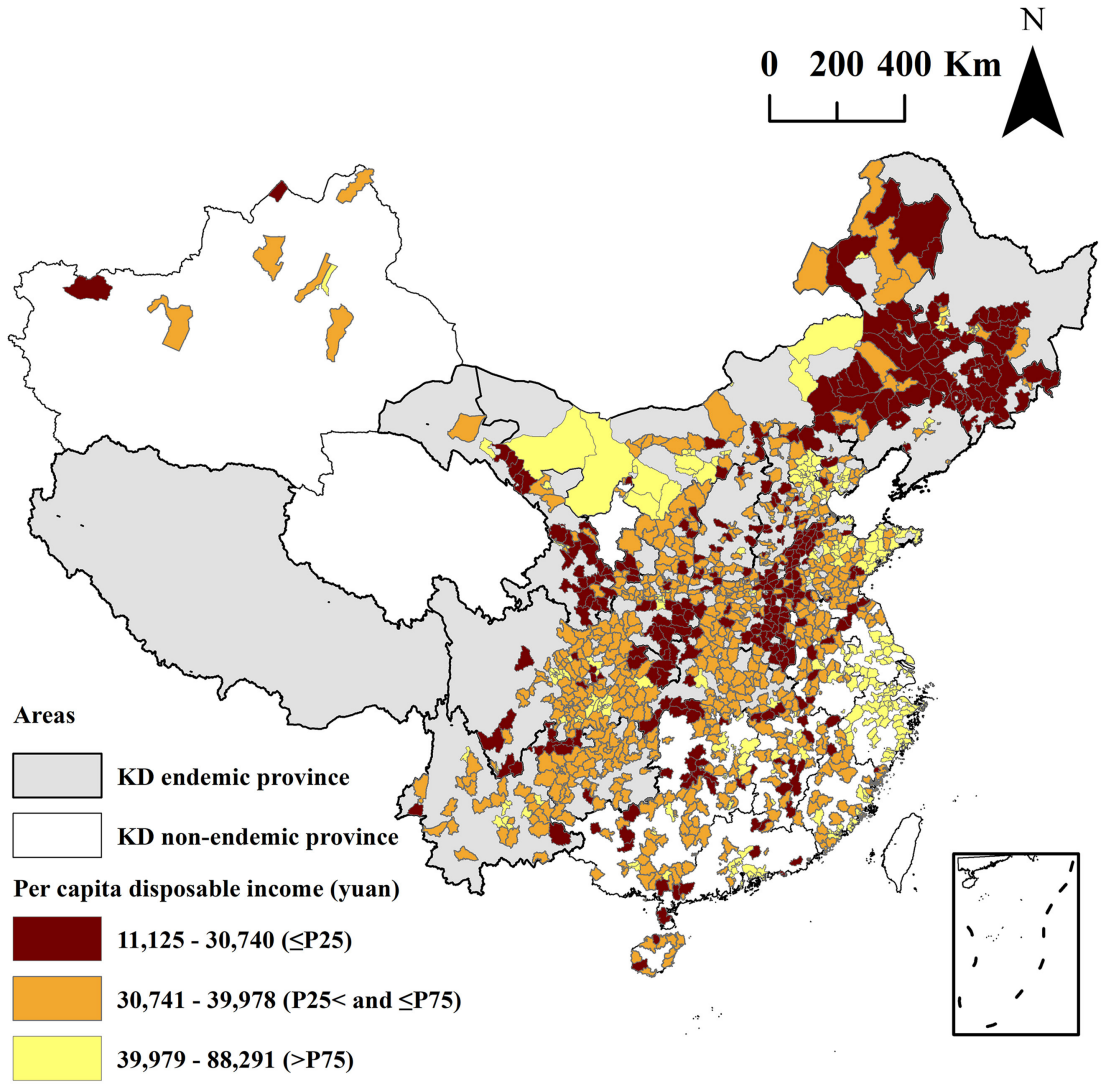
Spatial distribution of the mean per capita disposable income at the county-level.

The spatial distribution of the mean per capita disposable income in 1,333 counties are shown in Figure 3. The mean levels of the per capita disposable income in all 1,333 counties were 36,882 yuan, and ranged from 11,125 to 88,291 yuan. The P_25_ and P_75_ value of the per capita disposable income in 1,333 counties were 30,740 and 39,978 yuan, respectively. A total of 334 counties of the per capita disposable income were lower than the P_25_ value. Among them, 88 counties of the mean serum SELENOP levels and per capita disposable income were both lower than the P_25_ value of serum SELENOP and per capita disposable income. The mean levels of the per capita disposable income in KD endemic counties and KD non-endemic counties were 32,184 and 37,546 yuan, respectively. The mean levels of the per capita disposable income in KD endemic counties were significantly lower than that of per capita disposable income in KD non-endemic areas (*z* = 10.42, *P* < 0.0001).

A spatial regression model was developed with the serum SELENOP level as the dependent variable and per capita disposable income as the independent variable. The results of the spatial regression model were significant (*F* = 12.37, *P* = 0.0004). Finally, the serum SELENOP level was positively correlated with per capita disposable income (*t* = 3.52, *P* = 0.0004), as shown in Table 2.

**Table 2:** Spatial regression analysis of the serum SELENOP and per capita disposable income.

**Characteristic**	**Regression coefficients**	**Standard deviation**	***t***	***P-*value**
**β_0_^a^**	4.034083	0.116618	34.59	<0.0001
Per capita disposable income	0.000011	0.000003	3.52	0.0004

a *β_0_ is a constant term, R^2^ = 0.0094, Radj^2^ = 0.0086*.

### Spatial Autocorrelation Analysis

**Figure 4:**
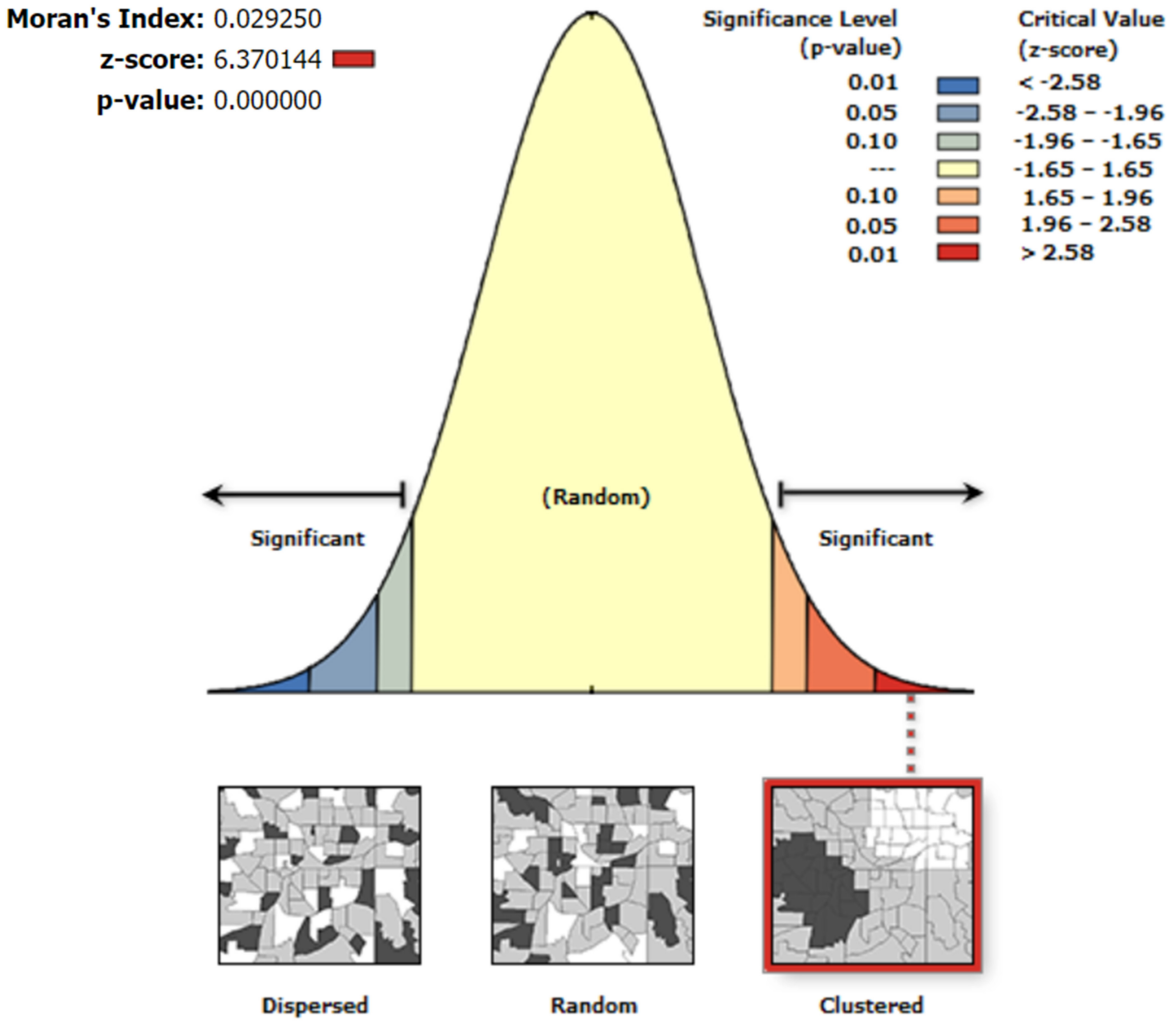
Global spatial autocorrelation analysis of the mean serum SELENOP levels at the county-level. The left side of the figure represents dispersed areas, the right side represents clustered areas, and the middle represents random areas.

The results of the global autocorrelation analysis of the mean serum SELENOP levels of the residents in 1,668 counties were significant (Moran's *I* = 0.03, *z* = 6.37, and *P* < 0.0001), indicating that the mean serum SELENOP levels in different counties had a spatially positive autocorrelation and was globally clustered at the overall level. The details are shown in Figure 4.

**Figure 5:**
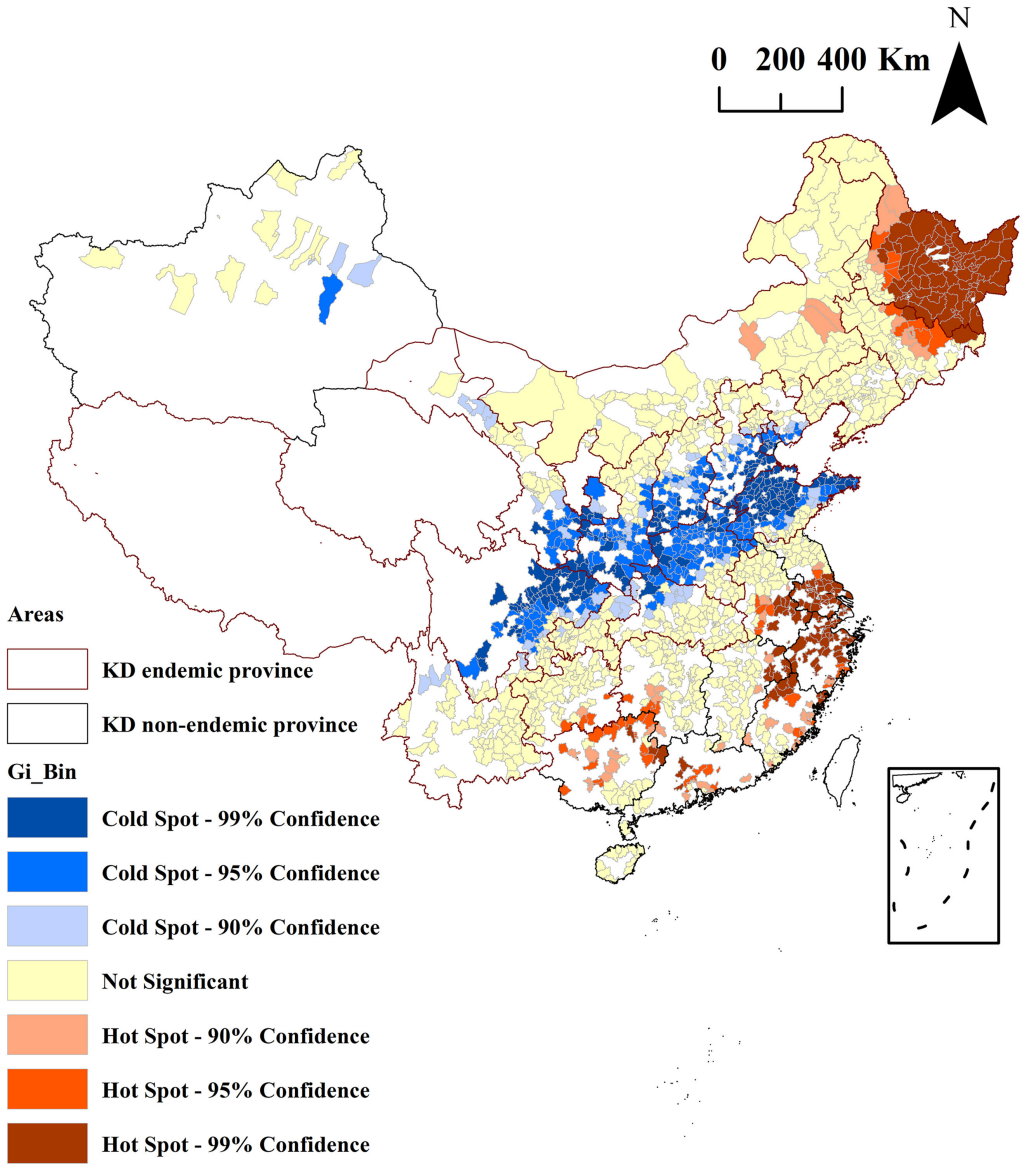
Clusters identified by local Getis-Ord Gi* analysis for the mean serum SELENOP levels by county in China. Red borders in the spatial thematic map represent KD-endemic areas. Colors in the spatial thematic map represent hot spots and cold spots of spatial clustering with 90, 95, and 99% CI.

The results of the Getis-Ord-Gi^*^ analysis of the mean serum SELENOP levels of the residents in 1,668 counties are shown in Figure 5. The cold spots areas were blue in color, indicated that the low values of the mean serum SELENOP levels were clustered in these areas. The hot spots areas were red in color, indicated that the high values of the mean serum SELENOP levels were clustered in these areas. The areas not spatially clustered were yellow in color. According to the 90, 95, and 99% CI of Getis-Ord-Gi^*^ statistic, the cold spots were 213, 248, and 96 counties, respectively; and the hot spots were 67, 65, and 218 counties, respectively. Of the 557 cold spots areas of low serum SELENOP levels, 553 (99.3%, 553/557) counties were located in 11 KD endemic provinces of Yunnan, Sichuan, Gansu, Chongqing, Shaanxi, Shanxi, Hubei, Henan, Hebei, Shandong, and Inner Mongolia; only 4 (0.7%, 4/557) counties were located in KD non-endemic province of Xinjiang. Of the 350 hot spots areas of high serum SELENOP levels were located in 4 KD endemic provinces of Guizhou, Heilongjiang, Jilin, and Inner Mongolia and in 8 KD non-endemic provinces of Guangxi, Hunan, Guangdong, Jiangxi, Fujian, Anhui, Zhejiang, and Jiangsu.

## Discussion

This was the first nationwide spatial ecological study on SELENOP and KD at the county-level, which is small area study. The smaller the spatial unit of the study, the better the homogeneity of the units, the more stable the output model, the more precise the visualization, and the more reliable and accurate the results.

Assessment of KD elimination has been being the first priority of KD prevention and control in the past 10 years. Selenium deficiency is the most recognized and convincing cause on the KD etiological aspects (10–12). SELENOP, accounting for about 60% of total plasma selenium in the human body, is a recognized molecular biomarker of selenoproteins and an important indicator of selenium nutrition (23, 30). It is essential to conduct the county-level spatial ecological study of serum SELENOP and KD. A total of 6,382 individuals were recruited from 1,688 counties in 29 provinces, and covered 59.4% (1,688/2,843) of all counties in mainland China, including 236 KD endemic counties (71.5%, 236/330) and 1,452 KD non-endemic counties (57.8%, 1,452/2,513) in mainland China. The scope of the study is wide.

As shown in Table 1, the mean serum SELENOP levels of the 6,382 residents in 1,688 counties were 4.62 ± 1.82 μg/mL. These seemed lower than the human serum SELENOP concentration (5.4 ± 0.5 μg/mL) reported by Hybsier et al. and higher than 4.3 μg/mL of a large prospective cohort investigation of 1,932 healthy Europeans reported by Hughes et al. (31, 32). Our research provides a useful reference for the definition of SELENOP reference values in humans, and still provides valuable information for assessing the effectiveness of KD prevention on the etiological aspects of selenium nutrition at the molecular level.

The mean serum SELENOP levels of residents living in the townships and rural areas of KD endemic counties were not statistically significantly lower than those of residents living in KD non-endemic counties. This is consistent with the low KD prevalence found in KD elimination assessment so this can be the evidence of KD elimination on the etiological perspective. However, the mean serum SELENOP levels in KD endemic counties were statistically significantly lower than KD non-endemic counties. The reason for this result is that the mean serum SELENOP levels of participants living in the urban centers of KD endemic counties were significantly lower than that of participants living in KD non-endemic counties. Furthermore, the mean serum SELENOP levels of residents living in rural areas and townships were significantly lower than those of residents living in the urban centers. These results indicated that selenium deficiency may still exist among residents living in some KD endemic areas, and they are still at risk having KD. The human body intakes selenium mainly through food, and the selenium content of food extremely is influenced by external environmental selenium level (33). It has been well-demonstrated that the selenium levels of external environments (soil and foods) of the residents living in KD endemic areas had been low (7–9) and low selenium levels of the foods eventually leaded to selenium deficiency in the residents of the KD endemic areas (33, 34). Therefore, the diets of foods rich in selenium should be still recommended for the residents of the KD endemic areas.

As shown in Figures 2, 3, the mean serum SELENOP levels and per capita disposable income of 88 counties out of 1,688 surveyed counties were low. This result was not only consistent with the result that the serum SELENOP level was positively correlated with per capita disposable income (*t* = 3.52, *P* = 0.0004) in the spatial regression model but also consistent with the result that the KD prevalence was negatively correlated with per capita disposable income in the KD prevalence assessment (35). Furthermore, the mean levels of the per capita disposable income in KD endemic counties were significantly lower than in KD non-endemic areas (*z* = 10.42, *P* < 0.0001). This result was consistent with the result that the mean serum SELENOP levels of residents living in rural areas and townships were significantly lower than those of residents living in the urban centers because KD endemic areas are all located in rural areas. These results indicated that serum SELENOP levels were closely correlated with economic factors.

As shown in Figure 4, the results of the global autocorrelation analysis of the mean serum SELENOP levels of the residents in 1,668 counties were significant (Moran's *I* = 0.03, *z* = 6.37, and *P* < 0.0001), indicating that the mean serum SELENOP levels were globally clustered at the overall level and had a positive correlation between serum SELENOP levels in counties and their neighboring counties. Although global spatial autocorrelation analysis can explore whether there is spatial clustering of serum SELENOP levels at the overall level, it cannot identify the areas and types of spatial clusters. Thus, local spatial autocorrelation analysis is essential. Getis-Ord Gi^*^ is a commonly used indicator for evaluating local autocorrelation. As shown in Figure 5, Getis-Ord-Gi^*^ statistic effectively identified the cluster areas. Of the 557 cold spots areas of low serum SELENOP levels, 553 (99.3%, 553/557) counties were located in 11 KD endemic provinces of Yunnan, Sichuan, Gansu, Chongqing, Shaanxi, Shanxi, Hubei, Henan, Hebei, Shandong, and Inner Mongolia; only 4 (0.7%, 4/557) counties were located in KD non-endemic province of Xinjiang. These results can be the geographically precisely visualized evidence of selenium nutrition at molecular level for assessing KD prevention, control, and elimination. The clustered areas of cold spots in the KD endemic provinces should be the target for the high priority of KD precision prevention and control. The majority of hot spots clustered areas of high serum SELENOP levels were located in Heilongjiang province, in which KD was severely epidemic in the 1950s through the early 1970s. There are 66 KD endemic counties in Heilongjiang province, accounting for 20% (66/330) of all KD endemic counties in China. This result was not very consistent with the results that the serum SELENOP level was positively correlated with per capita disposable income. In China, the per capita disposable income and per capita gross domestic product (GDP) of Heilongjiang ranked almost at the bottom in 2019 (36). Further studies are needed clarify this issue.

The major innovations of this study are that firstly this was the first nationwide county-level spatial ecological study of serum SELENOP and KD. SELENOP is a recognized molecular biomarker of selenium nutrition. This study is therefore a molecular spatial epidemiological study having significance in public health for primary prevention. Secondly, the county-level small area study, a higher geographically precisely visualized technique and stronger scientific in spatial epidemiological analysis, were more reliable and geographically precise. The findings of this study were not only the molecular evidence of serum SELENOP for assessing KD prevention, control, and elimination at county-level on the etiological aspects but also geographically precise and visualized evidence for the strategic plans for KD prevention and control. Ultimately, the innovation of this study is the translation of SELENOP and the techniques of small area study of spatial epidemiology into the practice of KD precision prevention and control. The limitations of this study were that the study was not probability sampling, and the sample sizes of some counties were limited. Another limitation of this study is the lack of total serum selenium measurements, even though SELENOP and total serum selenium displayed a significant correlation. SELENOP, a primary selenium transport protein and reflects long-term intake that is less influenced by the chemical form of the ingested selenium, is regarded as the most meaningful and the best functional biomarker of selenium nutritional status (21, 32). However, there seems to be no correlation between SELENOP and selenium when selenium concentration below 50–60 μg/L in serum or plasma. In such case, the SELENOP may not reflect the selenium status in a proper way and therefore total serum or plasma selenium is regarded as the most reliable biomarker and total selenium would considerably strengthen the study. An additional limitation of the study is lack of validation of SELENOP ELISA assay by measuring certified reference material to get a more precise quantification of SELENOP in human blood.

In conclusion, the mean serum SELENOP levels of the residents living in the rural areas of KD endemic counties were not statistically significantly lower than those of the residents living in KD non-endemic counties. This is consistent with the low KD prevalence found in KD elimination assessment so this can be the evidence of KD elimination on the etiological perspective. The mean serum SELENOP levels were globally clustered at the overall level. The majority of cold spots clustered areas identified by the Getis-Ord-Gi^*^ analysis are in the KD endemic provinces. These counties should be the geographically precisely visualized evidence for the high priority of KD precision prevention and control.

## Data Availability Statement

The raw data supporting the conclusions of this article will be made available by the authors, without undue reservation.

## Ethics Statement

The studies involving human participants were reviewed and approved by the Ethics Committee of the Harbin Medical University (hrbmuecdc20150101). All participants provided written informed consent. Written informed consent from the participants' legal guardian/next of kin was not required to participate in this study in accordance with the national legislation and the institutional requirements.

## Author Contributions

TW, QL, YaW, and YJ contributed to the study conception and design. YJ, RW, SS, LQ, YuW, YZo, XL, YZh, JH, HF, QL, and TW performed material preparation, data collection, and analysis. YJ and TW wrote the first draft of the manuscript. All authors commented on previous versions of the manuscript and read and approved the final manuscript.

## Funding

This work was supported by the National Natural Science Foundation of China (Grant Numbers 82073492, 81773368, 81202154, and 81172607).

## Conflict of Interest

The authors declare that the research was conducted in the absence of any commercial or financial relationships that could be construed as a potential conflict of interest.

## Publisher's Note

All claims expressed in this article are solely those of the authors and do not necessarily represent those of their affiliated organizations, or those of the publisher, the editors and the reviewers. Any product that may be evaluated in this article, or claim that may be made by its manufacturer, is not guaranteed or endorsed by the publisher.

## References

[1] The Ministry of Health of the People's Republic of China. The Criteria for Diagnosis of Keshan Disease (WS/T 210-2011). (2011). Available online at: http://www.nhc.gov.cn/ewebeditor/uploadfile/2013/01/20130109165522244.pdf (accessed September 20, 2021).

[2] Li GS Wang F Kang D Li C. Keshan disease: an endemic cardiomyopathy in China. Hum Pathol. (1985) 16:602–9. 10.1016/S0046-8177(85)80110-6 3997137

[3] Hou J Zhu LF Chen CC Feng HQ Li DD Sun SQ. Association of selenium levels with the prevention and control of Keshan disease: A cross-sectional study. J Trace Elem Med Biol. (2021) 68:126832. 10.1016/j.jtemb.2021.126832 34364066

[4] Li Q Liu MF Hou J Jiang CX Li SC Wang T. The prevalence of Keshan disease in China. Int J Cardiol. (2013) 168:1121–6. 10.1016/j.ijcard.2012.11.046 23218571

[5] Wang T. Assessment of Keshan disease elimination: the challenges and opportunities. Chin J Endemiol. (2015) 34:391–2. 10.3760/cma.j.issn.2095-4255.2015.06.001

[6] Li SE Wang T Ye C Li Q Guo ZD Wu H. An approach to assessment of Keshan disease elimination at the township level. Int Health. (2016) 8:398–404. 10.1093/inthealth/ihw045 27821502

[7] Chen SX Yang GQ Chen JS Chen XC Wen ZM Ge KY. Studies on the relations of selenium and Keshan disease. Biol Trace Elem Res. (1980) 2:91–107. 10.1007/BF02798589 24272892

[8] Xu GL. The prevention of Keshan disease by sodium selenite and the relationship between selenium deficiency and Keshan disease. J Xi'an Jiaotong Univ. (1987) 8:329–33.

[9] Loscalzo J. Keshan disease, selenium deficiency, and the selenoproteome. N Engl J Med. (2014) 370:1756–60. 10.1056/NEJMcibr1402199 24785212

[10] Yang GQ Wang GY Yin TA. Relationship between Keshan disease distribution and selenium nutrition condition in China. Acta Nutr Sinica. (1982) 4:191–200.

[11] Xia YM Hill KE Burk RF. Effect of selenium deficiency on hydroperoxide-induced glutathione release from the isolated perfused rat heart. J Nutr. (1985) 115:733–42. 10.1093/jn/115.6.733 3998867

[12] Xia YM Hill KE Burk RF. Biochemical studies of a selenium-defificient population in China: measurement of selenium, glutathione peroxidase and other oxidant defense indices in blood. J Nutr. (1989) 119:1318–26. 10.1093/jn/119.9.1318 2795246

[13] Zhou HH Wang T Li Q Li DD. Prevention of Keshan disease by selenium supplementation: a systematic review and meta-analysis. Biol Trace Elem Res. (2018) 186:98–105. 10.1007/s12011-018-1302-5 29627894

[14] Müller SM Dawczynski C Wiest J Lorkowski S Kipp AP Schwerdtle T. Functional biomarkers for the selenium status in a human nutritional intervention study. Nutrients. (2020) 12:676. 10.3390/nu12030676 32131476 PMC7146433

[15] Ekoue DN Zaichick S Valyi-Nagy K Picklo M Lacher C Hoskins K. Selenium levels in human breast carcinoma tissue are associated with a common polymorphism in the gene for SELENOP (Selenoprotein P). J Trace Elem Med Biol. (2017) 39:227–233. 10.1016/j.jtemb.2016.11.003 27908419

[16] Saito Y. Selenium transport mechanism via selenoprotein P-its physiological role and related diseases. Front Nutr. (2021) 8:685517. 10.3389/fnut.2021.685517 34124127 PMC8193087

[17] Read R Bellew T Yang JG Hill KE Palmer IS Burk RF. Selenium and amino acid composition of selenoprotein P, the major selenoprotein in rat serum. J Biol Chem. (1990) 265:17899–905. 10.1016/S0021-9258(18)38248-6 2211667

[18] Burk RF Hill KE. Selenoprotein P: an extracellular protein with unique physical characteristics and a role in selenium homeostasis. Annu Rev Nutr. (2005) 25:215–35. 10.1146/annurev.nutr.24.012003.132120 16011466

[19] Hill KE Xia YM Akesson B Boeglin ME Burk RF. Selenoprotein P concentration in plasma is an index of selenium status in selenium-deficient and selenium-supplemented Chinese subjects. J Nutr. (1996) 126:138–45. 10.1093/jn/126.1.138 8558294

[20] Xia YM Hill KE Li P Xu JY Zhou DY Motley AK. Optimization of selenoprotein P and other plasma selenium biomarkers for the assessment of the selenium nutritional requirement: a placebo-controlled, double-blind study of selenomethionine supplementation in selenium-deficient Chinese subjects. Am J Clin Nutr. (2010) 92:525–31. 10.3945/ajcn.2010.29642 20573787 PMC2921536

[21] Brodin O Hackler J Misra S Wendt S Sun Q Laaf E. Selenoprotein P as biomarker of selenium status in clinical trials with therapeutic dosages of selenite. Nutrients. (2020) 12:1067. 10.3390/nu12041067 32290626 PMC7230801

[22] Shetty S Marsicano JR Copeland PR. Uptake and utilization of selenium from selenoprotein P. Biol Trace Elem Res. (2018) 181:54–61. 10.1007/s12011-017-1044-9 28488249 PMC5680150

[23] Isobe Y Asakura H Tsujiguchi H Kannon T Takayama H Takeshita Y. Alcohol intake is associated with elevated serum levels of selenium and selenoprotein P in humans. Front Nutr. (2021) 8:633703. 10.3389/fnut.2021.633703 33693023 PMC7937717

[24] Graham AJ Atkinson PM Danson FM. Spatial analysis for epidemiology. Acta Trop. (2004) 91:219–25. 10.1016/j.actatropica.2004.05.001 15246928

[25] Karunaweera ND Ginige S Senanayake S Silva H Manamperi N Samaranayake N. Spatial epidemiologic trends and hotspots of leishmaniasis, Sri Lanka, 2001-2018. Emerg Infect Dis. (2020) 26:1–10. 10.3201/eid2601.190971 31855147 PMC6924882

[26] Sadeq M. Spatial patterns and secular trends in human leishmaniasis incidence in Morocco between 2003 and 2013. Infect Dis Poverty. (2016) 5:48. 10.1186/s40249-016-0135-8 27164836 PMC4863334

[27] Zhang X Wang T Li SE Ye C Hou J Li Q. A spatial ecological study of selenoprotein P and Keshan disease. J Trace Elem Med Biol. (2019) 51:150–8. 10.1016/j.jtemb.2018.10.011 30466925

[28] The Ministry of Health of the People's Republic of China. Delimitation and Classification of Keshan Disease Areas (GB17020-2010). (2011). Available online at: http://www.nhc.gov.cn/wjw/s9500/201106/51931/files/331adfa14f7e4f6a9a40c7e055fb4ba1.pdf (accessed September 20, 2021).

[29] The Ministry of Civil Affairs of the People's Republic of China. The Administrative Division of the People's Republic of China. (2020). Available online at: http://www.mca.gov.cn/ (accessed September 20, 2021).

[30] Wang YN Zou YJ Wang T Han S Liu X Zhang YY. A spatial study on serum selenoprotein P and Keshan disease in Heilongjiang Province, China. J Trace Elem Med Biol. (2021) 65:126728. 10.1016/j.jtemb.2021.126728 33610059

[31] Hybsier S Schulz T Wu ZD Demuth I Minich WB Renko K. Sex-specific and inter-individual differences in biomarkers of selenium status identified by a calibrated ELISA for selenoprotein P. Redox Biol. (2017) 11:403–14. 10.1016/j.redox.2016.12.025 28064116 PMC5220167

[32] Hughes DJ Fedirko V Jenab M Schomburg L Méplan C Freisling H. Selenium status is associated with colorectal cancer risk in the European prospective investigation of cancer and nutrition cohort. Int J Cancer. (2015) 136:1149–61. 10.1002/ijc.29071 25042282

[33] Vinceti M Filippini T Wise LA. Environmental selenium and human health: an update. Curr Environ Health Rep. (2018) 5:464–85. 10.1007/s40572-018-0213-0 30280317

[34] Skalny AV Burtseva TI Salnikova EV Ajsuvakova OP Skalnaya MG Kirichuk AA. Geographic variation of environmental, food, and human hair selenium content in an industrial region of Russia. Environ Res. (2019) 171:293–301. 10.1016/j.envres.2019.01.038 30708233

[35] Han XM Wang T Guo ZY Hou J Duan YN Wang YN. Analysis of spatial distribution characteristics of CKD in China. Chin J Endemiol. (2018) 37:301–5. 10.3760/cma.j.issn.2095-4255.2018.04.009

[36] National Bureau of Statistics. China Statistical Yearbook 2020. (2021). Available online at: http://www.stats.gov.cn/tjsj/ndsj/2020/indexch.htm (accessed October 29, 2021).

